# Combining Carcinoembryonic Antigen and Platelet to Lymphocyte Ratio to Predict Brain Metastasis of Resected Lung Adenocarcinoma Patients

**DOI:** 10.1155/2017/8076384

**Published:** 2017-05-31

**Authors:** Wei Wang, Chao Bian, Di Xia, Jin-Xi He, Ping Hai, Ren Zhao, Yan-Yang Wang

**Affiliations:** ^1^Department of Radiation Oncology, General Hospital of Ningxia Medical University, Yinchuan, Ningxia 750004, China; ^2^Cancer Institute, Ningxia Medical University, Yinchuan, Ningxia 750004, China; ^3^Graduate School, Ningxia Medical University, Yinchuan, Ningxia 750004, China; ^4^Department of Thoracic Surgery, General Hospital of Ningxia Medical University, Yinchuan, Ningxia 750004, China

## Abstract

We aimed to evaluate the role of pretreatment carcinoembryonic antigen (CEA) and platelet to lymphocyte ratio (PLR) in predicting brain metastasis after radical surgery for lung adenocarcinoma patients. The records of 103 patients with completely resected lung adenocarcinoma between 2013 and 2014 were reviewed. Clinicopathologic characteristics of these patients were assessed in the Cox proportional hazards regression model. Brain metastasis occurred in 12 patients (11.6%). On univariate analysis, N2 stage (*P* = 0.013), stage III (*P* = 0.016), increased CEA level (*P* = 0.006), and higher PLR value (*P* = 0.020) before treatment were associated with an increased risk of developing brain metastasis. In multivariate model analysis, CEA above 5.2 ng/mL (*P* = 0.014) and PLR ≥ 120 (*P* = 0.036) remained as the risk factors for brain metastasis. The combination of CEA and PLR was superior to CEA or PLR alone in predicting brain metastasis according to the receiver operating characteristic (ROC) curve analysis (area under ROC curve, AUC 0.872 versus 0.784 versus 0.704). Pretreatment CEA and PLR are independent and significant risk factors for occurrence of brain metastasis in resected lung adenocarcinoma patients. Combining these two factors may improve the predictability of brain metastasis.

## 1. Introduction

Lung cancer is the first cause of cancer death in the world, and adenocarcinoma is the most diagnosed histological subtype [1, 2]. Although surgical resection offers the best curative option for early stage lung adenocarcinoma, recurrence after surgery is still a critical problem, especially for brain metastasis, which is the major contribution to cancer death [3, 4]. In order to further improve the survival of early stage lung adenocarcinoma patients, prevention of the occurrence of brain metastasis is one of the treatment options. It is well-known that prophylactic cranial irradiation (PCI) has been used in the treatment of small cell lung cancer patients, which can reduce the frequency of brain metastasis and prolong the survival [5, 6]. However, the value of this approach in the management of non-small cell lung cancer (NSCLC) patients is still in doubt [7]. Hence, defining the predictive factors for brain metastasis development and identifying high risk lung adenocarcinoma patients who will benefit from PCI is meaningful [8–10].

Carcinoembryonic antigen (CEA) is an oncofetal protein attached to epithelial-cell apical membrane via its c-terminal glycosylphosphatidylinositol anchor, a member of the immunoglobulin superfamily of cell adhesion molecules (IgCAM) [11]. High serum CEA levels have been associated with brain metastasis development and poor prognosis in patients with advanced NSCLC [12–15]. However, as we know, the relationship between baseline serum CEA levels and the brain metastasis development in resected lung adenocarcinoma patients is still not clear.

Inflammation is increasingly recognized as being closely associated with cancer initiation and development. Inflammation can enhance tumor growth, invasion, angiogenesis, and, eventually, metastasis [16, 17]. Therefore, markers of inflammation may provide useful information for cancer diagnosis and management. The platelet to lymphocyte ratio (PLR), defined as the absolute platelet count divided by the absolute lymphocyte count, is a representative index of systemic inflammation. Its prognostic value has been studied in many types of cancers, including breast cancer, ovarian cancer, pancreatic cancer, and colorectal cancer [18–21]. Recent studies suggest a potential prognostic role of PLR in lung cancer patients [22–27]. However, to our knowledge, no research has evaluated the role of PLR in predicting brain metastasis development for lung adenocarcinoma patients.

In this study, we reviewed the patients with completely resected lung adenocarcinoma and aimed to identify the predictive role of CEA and PLR and the combination analysis of these two factors in metastasis to brain of the curatively resected lung adenocarcinoma patients.

## 2. Methods

### 2.1. Patient Population and Clinical Data Collection

The study included 103 patients with pathologically confirmed lung adenocarcinoma who had received complete resection at General Hospital of Ningxia Medical University from 2013 to 2014. Clinicopathologic information of these patients, including age, sex, smoking history, tumor location, histological grade, tumor size, lymph node metastasis, TNM stage, and postoperative treatment modalities, was obtained from electronic medical records. TNM stage was classified according to the UICC/AJCC 7th TNM staging system, published in 2009 [28]. The pretreatment CEA and hemoglobin level were also included in the analysis. The PLR was defined as the absolute platelet count divided by the absolute lymphocyte count. Patients who were previously diagnosed with cancer other than nonmelanomatous skin cancer and who did not undergo brain computed tomography (CT) scans or magnetic resonance imaging (MRI) as part of their preoperative staging procedure were excluded from the study. And also, patients with hematologic, autoimmune, or infectious diseases or who received preoperative anticancer therapy were excluded. This study was approved by the ethics committee of General Hospital of Ningxia Medical University (number 2016-198).

### 2.2. Treatment and Follow-Up

All of the patients received complete pulmonary resection and systematic node dissection of the ipsilateral hilar and mediastinal lymph nodes. The patients with stage IB or higher lung cancer were given postoperative adjuvant therapy according to the National Comprehensive Cancer Network (NCCN) guidelines.

The follow-up was defined from the time of pulmonary resection. Physical examination, complete blood test, enhanced CT for chest, and ultrasound examination for abdomen were performed every 6 months for 2 years. Contrast enhanced CT or MRI of the brain was performed if brain metastasis was suspected or yearly. Disease progression and failure sites were determined by radiologic examination, histologic examination, or both. The median follow-up time of the whole study population was 30 months (range, 4–42 months).

### 2.3. Statistical Analysis

All continuous variables were dichotomized to categorical variables basis on the median values of the sample. Potential risk factors of developing brain metastasis were evaluated by univariable and multivariable Cox proportional hazard model. The area under the curve (AUC) was used to assess the predictive value of each risk factor. Brain metastasis free survival time was defined as the period from date of the pulmonary resection to brain metastasis or the last follow-up. The brain metastasis free survival curves were calculated according to the Kaplan-Meier method with the log-rank test. All reported *P* values were two-sided, and *P* less than 0.05 was considered statistically significant. SPSS 13.0 (SPSS Inc., Chicago, IL) was used for the statistical analysis.

## 3. Results

Among the 103 patients included in the study, the median age was 61 years (range, 36 to 79 years), and 61.2% (63 cases) were female. 41.7% (43 cases) of these patients had smoking history. The majority of tumors (52.4%) are located in the upper lobe. With regard to histological grade in the 103 patients, 85.4% (88 cases) were well-moderate differentiated grade and 14.6% (15 cases) were poorly differentiated. According to the UICC/AJCC 7th TNM staging system, 63 (61.2%) patients were stages I and II and 40 (38.8%) patients were stage III. Majority of these patients (77.7%) received adjuvant therapy after pulmonary resection. Based on the threshold value of our hospital, patients were divided into two groups according to pretreatment CEA value (≥5.2 ng/mL and <5.2 ng/mL) or hemoglobin level (≥115 g/L and <115 g/L). According to the results of receiver operating characteristic (ROC) curve analysis, the enrolled patients were also divided into two groups by the median value of PLR (≥120 and <120). The summary of patients' characteristics are shown in Table 1. For the whole study population, the median follow-up from the time of surgery was 30 months. At the end of follow-up, 10 patients (9.7%) developed local and regional recurrence. Metastasis to the brain, bone, liver, and lung occurred in 12 (11.6%), 6 (5.8%), 6 (5.8%), and 3 (2.9%) patients, respectively. The median number of the brain metastasis lesions was 3 (range, 1–16). The median time of the brain metastasis development was 12 months (range, 4–22 months).

Several clinical and pathological factors were found to be associated with the brain metastasis of resected lung adenocarcinoma patients on both univariate and multivariate analyses. In univariate analysis, N2 stage (*P* = 0.013), stage III (*P* = 0.016), increased CEA level (*P* = 0.006), and higher PLR value (*P* = 0.020) before treatment were associated with an increased risk of developing brain metastasis. Using all of these four high risk factors, we constructed a multivariate Cox proportional hazards regression model. In multivariate model analysis, CEA above 5.2 ng/mL (*P* = 0.014) and PLR ≥ 120 (*P* = 0.036) remained as the risk factors for brain metastasis (Table 2). In addition, the relationship between increased CEA level and tumor, patient, or metastatic characteristics was assessed. The results demonstrated that increased CEA level was associated with smoke history (*P* < 0.001), poorly differentiated histology grade (*P* = 0.005), and higher TNM stage (*P* = 0.011).

**Figure 1:**
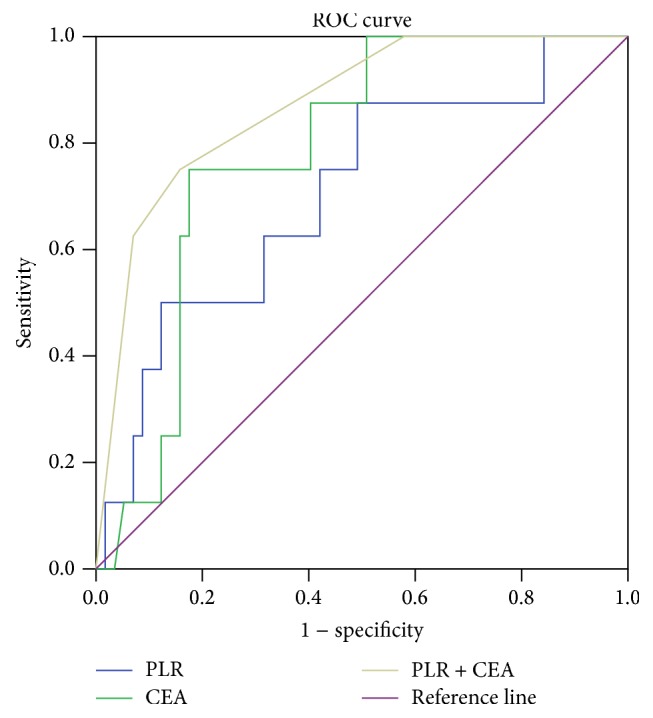
Receiving operator characteristic curve based on the sensitivity and specificity of CEA alone, PLR alone, or CEA and PLR combined.

**Figure 2:**
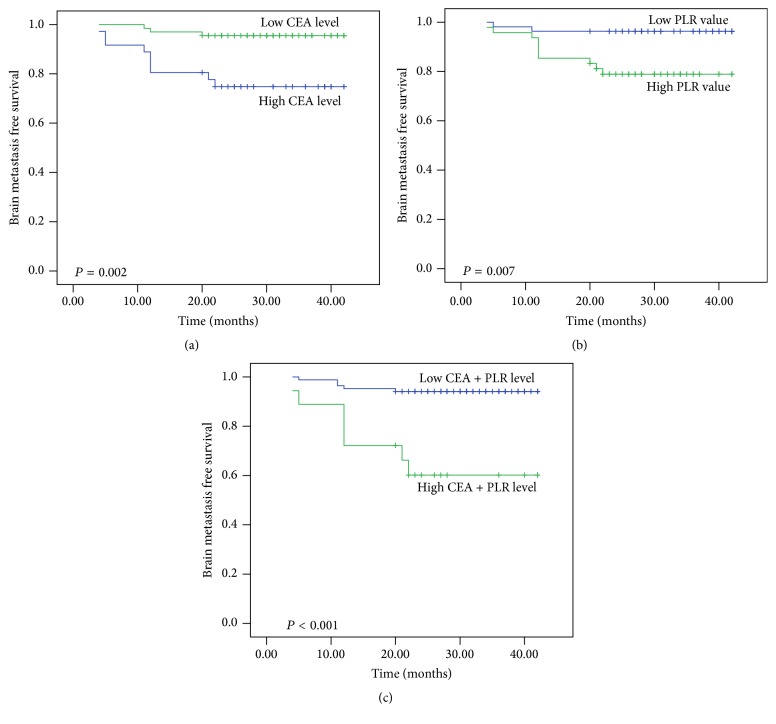
Brain metastasis free survival according to CEA alone (a), PLR alone (b), or CEA and PLR combined (c).

A new cut-off value, 15.6 ng/mL, was determined by ROC analysis for CEA. The ROC curves were generated for increased CEA (≥15.6 ng/mL) or higher PLR (≥120) and the combined analysis of these two factors. The combination of these two factors was superior to CEA alone or PLR alone in predicting brain metastasis according to the ROC analysis (AUC 0.872 versus 0.784 versus 0.704; Figure 1). These results indicated the potential predictive value of combined analysis of CEA and PLR in brain metastasis development of radical resected lung adenocarcinoma patients. The brain metastasis free survival curves, which are stratified by CEA, PLR, and the combination of these two factors, are shown in Figure 2.

## 4. Discussion

The majority of postoperative recurrences of NSCLC are distant metastasis, especially for brain metastasis [3]. Despite the advances in the treatment modalities [29–31], brain metastasis remains a major cause of mortality in patients with NSCLC. PCI has been investigated as a strategy to reduce the risk of brain metastasis for lung cancer patients. Several trials have shown that PCI is effective in reducing brain metastasis for NSCLC patients; however, there is no survival advantage [7]. The effort to identify lung cancer patients with high risk for developing brain metastasis would be able to avoid excessive treatment for patients with less aggressive tumors and increase the benefits of PCI [8, 9]. In addition, the identification of high risk patients for developing brain metastasis could also be used to establish an optimal follow-up strategy for detecting brain relapse. Until now, brain CT or MRI is not routinely used in the follow-up of postoperative NSCLC patients. However, more and more evidences suggest that early detection of brain metastasis by brain CT or MRI during follow-up may improve the survival of lung cancer patients [32, 33]. As such, in this study, we focus on the identification of predictors for developing brain metastasis in resected lung adenocarcinoma patients, who are more likely to suffer brain metastasis.

In present study, 11.6% of the patients had developed brain metastases by 30 months of follow-up. In univariate analysis, N2 stage, stage III, increased CEA level, and higher PLR value were associated with an increased rate of brain metastasis. However, in multivariable analysis, only increased CEA level and higher PLR value were selected as the predictors for the probability of developing brain metastasis after curative surgery in lung adenocarcinoma patients.

CEA, a type of *β*-1 glycoprotein and a member of the IgCAM superfamily, is produced by the CEACAM5 gene and expressed during the early fetal life [11]. In the previous study, some groups had reported the association between pretreatment serum CEA level and the brain metastasis development of NSCLC. Lee et al. [15] found that the pretreatment serum CEA level was significantly correlated with brain metastasis in advanced NSCLC. The AUC of serum CEA for the prediction of brain metastasis was 0.724. Arrieta et al. [13] studied 293 patients with NSCLC in IIIB-IV clinical stage in a prospective manner. They indicated that high CEA serum level (≥40 ng/mL) was a risk factor for brain metastasis development and was associated with poor prognosis in patients with advanced NSCLC. In current study, our findings also suggested that abnormal pretreatment serum CEA level was strongly correlated with increased brain metastatic potential in resected lung adenocarcinoma patients.

Inflammation is a hallmark of cancer [34]. More and more evidences show that the systemic inflammatory response is related to the initiation and progression of various forms of cancer [16, 17]. PLR, the relative value of a combined platelet and lymphocyte counts, is a promising prognostic inflammation marker. Although the mechanisms underlying the association of PLR and prognosis of NSCLC are still incompletely understood, the relationship of PLR and the prognosis of NSCLC was explored in several studies [22, 24–27]. One of the aims of the current study was to evaluate the value of the PLR in brain metastasis prediction. To our knowledge, this is the first study to investigate the association between the PLR and brain metastasis of NSCLC patients. The results of our study showed that higher pretreatment PLR value was a predictor of brain metastasis. Additionally, in the ROC analysis, the results demonstrated that AUC was 0.784 for CEA, 0.704 for PLR, and 0.872 for the combined analysis, indicating that the combination analysis was superior to CEA or PLR alone as a predictive factor in patients with lung adenocarcinoma who received complete resection.

As a retrospectively analysis, our study had some limitations. Firstly, the relatively small sample size may introduce the selection bias. Furthermore, due to the limited number of patients who had undergone EGFR status detection at the time of this study, the impact of EGFR status on the development of brain metastasis was not investigated in this study.

## 5. Conclusion

The present study demonstrates that increased CEA level and higher PLR value are independent risk factors for brain metastasis development of resected lung adenocarcinoma patients. Combined analysis CEA and PLR could improve the prediction efficacy of brain metastasis for completely resected lung adenocarcinoma patients. However, prospectively conducted studies are warranted to validate the results.

## Figures and Tables

**Table 1:** Clinicopathologic characteristics of 103 lung adenocarcinoma patients.

Characteristics	Number of patients	%
Age, years		
≥60	58	56.3
<60	45	43.7
Sex		
Male	40	38.8
Female	63	61.2
Smoking status		
Never	60	58.3
Ever	43	41.7
Tumor location		
Upper lobe	53	52.4
Nonupper lobe	60	47.6
Histology grade		
Well-moderate	88	85.4
Poor	15	14.6
T stage		
T1-2	86	83.5
T3-4	17	16.5
N stage		
N0-1	75	72.8
N2	28	27.2
TNM stage		
I-II	63	61.2
III	40	38.8
Adjuvant therapy		
Yes	80	77.7
No	23	22.3
CEA		
≥5.2 ng/mL	36	40.0
<5.2 ng/mL	67	60.0
Hb		
≥115 g/L	92	89.3
<115 g/L	11	10.7
PLR		
≥120	51	49.5
<120	52	50.5

CEA, carcinoembryonic antigen; Hb, hemoglobin; PLR, platelet to lymphocyte ratio.

**Table 2:** Univariate and multivariate Cox regression analyses estimating the risk factors of brain metastases of resected lung adenocarcinoma patients.

Clinicopathological factors		Univariable analysis			Multivariable analysis		
		Hazard ratio	95% CI	*P* value	Hazard ratio	95% CI	*P* value
Age, years	≥60 versus <60	1.514	0.456–5.029	0.498			
Gender	Male versus female	0.616	0.199–1.910	0.402			
Smoking status	Ever versus never	1.230	0.333–4.542	0.756			
Tumor location	Upper lobe versus nonupper lobe	0.698	0.222–2.200	0.539			
Histology grade	Well versus poor	0.873	0.263–2.898	0.824			
T stage	T3-4 versus T1-2	2.593	0.780–8.615	0.120			
N stage	N2 versus N0-1	4.304	1.365–13.575	0.013	1.374	0.283–6.669	0.693
TNM stage	III versus I-II	4.976	1.346–18.390	0.016	2.640	0.440–15.829	0.288
Adjuvant therapy	Yes versus no	1.175	0.318–4.343	0.808			
CEA	<5.2 ng/mL versus ≥5.2 ng/mL	0.162	0.004–0.598	0.006	0.194	0.052–0.722	0.014
Hb	≥115 g/L versus <115 g/L	0.510	0.111–2.330	0.358			
PLR	≥120 versus <120	6.085	1.333–27.285	0.020	5.149	1.117–23.729	0.036

CI, confidence interval; CEA, carcinoembryonic antigen; Hb, hemoglobin; PLR, platelet to lymphocyte ratio.
